# The Crucial Role of Intensive Care Surveillance After Transcatheter Tricuspid Interventions

**DOI:** 10.1016/j.jaccas.2026.109512

**Published:** 2026-08-19

**Authors:** Muhammad Junaid Mustafa, Muhammad Hassan Iqbal

**Affiliations:** Punjab Institute of Cardiology, Lahore, Punjab, Pakistan

The case report presented by Cooper et al^1^ provides valuable insight for managing acute right ventricular failure after transcatheter tricuspid valve replacement that compels us to read with great interest. This case highlights the need for surveillance for hemodynamic shifts after the EVOQUE system was used for severe tricuspid regurgitation.

The authors accurately and precisely illustrate acute afterload mismatch that may arise after correction of defective tricuspid valve in a patient with underlying pulmonary hypertension. This report provides several valuable clinical insights that may help clinicians, including the following:

• The necessity of extending intensive care unit surveillance over early-discharge mindset, as this patient's hemodynamic collapse began 20 hours post procedure and peaked at 30 hours.
• The successful transition to vasopressin from norepinephrine alongside dobutamine and inhaled epoprostenol, which provides a brilliant, physiology-driven management algorithm.^2^
• Highlighting the tricuspid annular plane systolic excursion:pulmonary artery systolic pressure ratio, which gives clinicians a noninvasive, accessible tool to screen for baseline right ventricular–pulmonary arterial uncoupling during preprocedural workups.

In this evolving field, we may encounter such clinical cases in which there is a lack of proper management protocols, and such cases provide valuable information that proves to be beneficial. Cooper and colleagues^1^ successfully move the transcatheter tricuspid valve replacement narrative beyond simple technical feasibility and into the critical realm of advanced perioperative care. Their work contributes to the establishment of safer, more comprehensive perioperative management strategies.

### Use of AI

The authors took help from AI but paragraphs are written by the authors in their own words.
